# Explaining changes in child health inequality in the run up to the 2015 Millennium Development Goals (MDGs): The case of Zambia

**DOI:** 10.1371/journal.pone.0170995

**Published:** 2017-02-07

**Authors:** Peter Hangoma, Arild Aakvik, Bjarne Robberstad

**Affiliations:** 1 Centre for International Health, University of Bergen, Bergen, Norway; 2 Unit of Health Economics, School of Public Health, University of Zambia, Lusaka, Zambia; 3 Department of Economics, University of Bergen, Bergen, Norway; 4 Centre for Intervention Science in Maternal and Child Health, University of Bergen, Bergen, Norway; Centre Hospitalier Universitaire Vaudois, FRANCE

## Abstract

**Background:**

Child health interventions were drastically scaled up in the period leading up to 2015 as countries aimed at meeting the 2015 target of the Millennium Development Goals (MDGs). MDGs were defined in terms of achieving improvements in average health. Significant improvements in average child health are documented, but evidence also points to rising inequality. It is important to investigate factors that drive the increasing disparities in order to inform the post-2015 development agenda of reducing inequality, as captured in the Sustainable Development Goals (SDGs). We investigated changes in socioeconomic inequality in stunting and fever in Zambia in 2007 and 2014. Unlike the huge literature that seeks to quantify the contribution of different determinants on the observed inequality at any given time, we quantify determinants of changes in inequality.

**Methods:**

Data from the 2007 and 2014 waves of the Zambia Demographic and Health Survey (DHS) were utilized. Our sample consisted of children aged 0–5 years (n = 5,616 in 2007 and n = 12,714 in 2014). We employed multilevel models to assess the determinants of stunting and fever, which are two important child health indicators. The concentration index (CI) was used to measure the magnitude of inequality. Changes in inequality of stunting and fever were investigated using Oaxaca-type decomposition of the CI. In this approach, the change in the CI for stunting/fever is decomposed into changes in CI for each determinant and changes in the effect—measured as an elasticity—of each determinant on stunting/fever.

**Results:**

While average rates of stunting reduced in 2014 socioeconomic inequality in stunting increased significantly. Inequality in fever incidence also increased significantly, but average rates of fever did not reduce.

The increase in the inequality (CI) of determinants accounted for the largest part (42.5%) of the increase in inequality of stunting, while the increase in the effect of determinants explained 35% of the increase. The determinants with the greatest total contribution (change in CI plus change in effect) to the increase in inequality of stunting were mother’s height and weight, wealth, birth order, facility delivery, duration of breastfeeding, and maternal education.

For fever, almost all (86%) the increase in inequality was accounted for by the increase in the effect of determinants of fever, while the distribution of determinants mattered less. The determinants with the greatest total contribution to the increase in inequality of fever were wealth, maternal education, birth order and breastfeeding duration.

In the multilevel model, we found that the likelihood of a child being stunted or experiencing fever depends on the community in which they live.

**Conclusions:**

To curb the increase in inequality of stunting and fever, policy may focus on improving levels of, and reducing inequality in, access to facility deliveries, maternal nutrition (which may be related to maternal weight and height), complementary feeding (for breastfed children), wealth, maternal education, and child care (related to birth order effects). Improving overall levels of these determinants contribute to the persistence of inequality if these determinants are unequally concentrated on the well off to begin with.

## Introduction

Socioeconomic inequalities in childhood health have persisted, with children from poor households experiencing a disproportionately larger burden [1, 2]. This also implies that they may bear a larger share of later life consequences of childhood ill-health. Apart from increasing under-5 mortality rates, childhood ill-health negatively affects cognitive abilities, education attainment, later life income, and adult health [3–6]. This study focusses on two key measures of childhood ill-health, namely, stunting and fever.

Fever is a broad measure of ill-health which may signal a number of sicknesses, including malaria and bacterial as well as viral infections [7–9]. In children under the age of 5 years, high fever may also lead to seizures, brain damage or death [10]. Similarly, stunting is a useful measure of childhood nutrition and ill-health. It is characterized by children being shorter than well-nourished kids of the same age and it is a culmination of chronic malnutrition or exposure to other adverse shocks. The adverse shocks may include illness, both in-utero [11] and in early life [12].

Of particular concern is the fact that fever and stunting, either directly or indirectly, constitutes a large share of childhood morbidity and mortality in low and middle income countries [13]. Perhaps more concerning is the persistence of inequalities in childhood ill-health [2]. Such inequalities are undesirable for at least two reasons. First, since inequalities in childhood health are related to inequalities in determinants of health—such as parental socioeconomic status, and access to health care, clean water, improved housing, neighborhoods etc.—which are predominately beyond the control of the child, and sometimes even parents themselves, it is considered unfair for some children to suffer more health challenges than others as a result of being at the disadvantage in accessing these determinants. Inequalities in child health are mostly a result of unequal opportunities in accessing determinants of good health so that such inequalities may be considered unnatural, avoidable and illegitimate. Avoidable inequalities are therefore termed inequities.

Second, persistence in inequalities in childhood ill-health is a source of concern on grounds that disease (whether infectious or not) in some parts of the population may affect the whole population [14]. On a national level, health inequality may affect economic growth [15]. Moreover, by plunging already poor households into health spending and possible labor income losses, e.g., due to taking care of sick children, health inequalities may widen income inequalities. Widening income inequalities are not only bad in themselves but they may also be harmful to the health of everyone in society, irrespective of their socioeconomic status [16].

Against this backdrop, reducing inequality has been espoused as one of the goals of the post 2015 development agenda, the Sustainable Development Goals (SDG). This is noteworthy because inequality was not an explicit goal in the Millennium Development Goals (MDGs), the predecessor to the SDGs. Despite the fact that policy documents in the MDG period emphasized the importance of reducing inequality, which they argued was key to achieving the MDGs on health [17–19], there have been concerns that MDGs were not appropriate goals to drive the inequality agenda because they mainly focused on improving average health with little or no attention on how unequally the gains are distributed [20]. An evaluation of the progress in child health in the MDG period leading up to 2015 shows that, despite acceleration in global reductions in under-5 mortality and a steady increase in life saving interventions, substantial inequalities in these interventions and in child health within and across countries have persisted [21]. Given the multi-country nature of this evaluation, it remains unclear as to what factors could have been driving the persistence in inequality despite the substantial increase in life saving interventions. We use data from Zambia to understand inequalities in childhood ill-health. Zambia presents a unique opportunity in assessing inequality because of the recently conducted 2014 Demographic and Health Survey (DHS), a rich nationally representative household survey.

Zambia experienced sharp increases in a number of child health interventions in the MDG period [22, 23]. At the same time, child mortality substantially reduced [23, 24]. After remaining stubbornly high in the 1992–2001 period, under-5 mortality rate sharply declined in the 2001–2014 period, from 168 per 1000 live births to 75 per 1000 live births. In the 1992–2001 period, it only reduced from 191 to 168 per 1000 live births [24]. The incidence as well as prevalence of key childhood ill-health also declined [24]. Although inequalities in child mortality and ill-health have been documented [25], it is unclear how these inequalities evolved and what factors could have been driving these changes in the period leading up to 2015.

This paper uses large nationally representative household survey data from the Zambia Demographic and Health Survey (DHS) collected in 2007 and 2014 to examine changes in inequality in stunting and fever. Three objectives are pursued. First, and as a starting point, it explores the determinants of (factors associated with) stunting as well as fever and examines whether the community in which the child lives affects their health. Second, the paper investigates the significance of socioeconomic inequality in stunting and fever in 2007 and 2014 as well as whether or not the magnitude of inequality changed in any significant way over this period. The concentration index (CI) is used to quantify socioeconomic inequality in these measures of childhood ill-health.

Third, this paper examines how determinants of fever and stunting may explain changes in socioeconomic inequality as captured by the change in the CI over the 2007–2014 period. The change in the CI for stunting/fever is decomposed into the relative contribution of each determinant, which is further broken down into two components: changes in the CI of each determinant and changes in the effect of each determinant on stunting/fever, measured as an elasticity. By adding up the percentage contributions of each of the two components, we are able to look at the change in the CI of stunting/fever that was accounted for by changes in CI of determinants on one hand and changes in the effect (elasticity) of determinants on the other hand.

Our study directly relates to studies from Vietnam that attempted to decompose the change in the concentration index of height for age. These studies found that rising inequality in height for age between 1990 and 2010 were mainly accounted for by both the increase in inequality in wealth and its elasticity [26, 27]. The challenge with these studies is that they used data that does not contain a rich set of health variables. These missing variables may explain a significant portion of changes in inequality and may also confound the relationship between wealth and height for age. Other than height for age (or stunting), we are not aware of any study that has attempted to decompose changes in inequality of fever, as we do in this study.

Our study also relates to a rich literature that decomposes inequality in other dimensions of child health. As opposed to decomposing changes in inequality, such decompositions are only able to decompose a single concentration index and are thus not able to explain or quantify the sources of the observed change in two concentration indices that differ in time or space. Most studies that conduct decompositions over time have centered on explaining changes in average health and not changes in inequality [28].

This study also contributes to the literature that explores the effect of key determinants such as maternal education, wealth, maternal nutrition and other key covariates, on child health. Despite the fact that our estimates cannot be viewed as causal due to the cross sectional nature of our data, the rich set of covariates enables us to gain useful insight into the drivers of childhood ill-health.

In this paper, we say inequality to mean socioeconomic inequality in health as opposed to total inequality in health.

## Data

Data were obtained from the 2007 and the 2014 Demographic and Health Survey (DHS). For children under the age of 5 years, our final dataset consisted of 5,616 observations in 2007 and 12,714 in 2014. The large difference in the number of observations between the two periods was due to the fact that the sample size for the 2014 DHS was more than doubled in order to provide reliable estimates for rural and urban areas within provinces [24].

The DHS uses a two stage sampling design where in the first stage, enumeration areas (clusters) are selected with probability proportional to size. The second stage selects households. In each household, three questionnaires are administered to eligible members by trained enumerators. The three questionnaires are the household’s, woman’s and man’s questionnaires [24].

Child health information is captured in both the household’s and woman’s questionnaire. Since, we are interested in children under the age of 5 years, only women who had given birth within the five-year period preceding the relevant survey year were included. Using mother’s identification variable, we merged the household and women data files. Children with missing mother identification variable either due to the mother being absent during the survey or due to incomplete interview were not included in the analysis.

Fever was measured by asking the mother whether her child had any fever within the two weeks preceding the survey. On the other hand, stunting was defined as having a height for age z-score of less than 2 standard deviations of the reference population using the WHO 2006 growth standards. Anthropometric measures (height and weight) were measured by the interviewer during each survey. Using the *zscore06* package in Stata [29], we computed height for age (HAZ) for each child. Consistent with the DHS methodology, HAZ was set to missing if height, age, or sex was either missing or out of range. All HAZ scores less than or greater than 6 standard deviation where regarded as out of range and dropped from the analysis.

The wealth index was calculated using principal component analysis and is provided together with the DHS data. Observations were ranked using the raw wealth index for purposes of computing concentration indices. In the decomposition analysis however, we grouped observations into quartiles.

## Methods

### Determinants of stunting and fever

For each survey year and each outcome, we fit a two level random intercept (multilevel) regression model. The first level is for the individual (child) while the second level is the community (enumeration area or cluster) where the child lives. The model takes the form:

$$y_{ijt}=\alpha_{jt}+\beta \prime x_{ijt}+\varepsilon_{ijt}$$ (1)

$$\alpha_{jt}=\delta_{j}+\mu_{jt}$$ (2)

where *y*_*ijt*_ is a binary variable equal to one if the outcome (fever or stunting) for child *i* residing in community *j* in year *t* is true. *α*_*jt*_, is the random effect for community *j* in year *t*, with *δ*_*j*_ being the time average random effect for community *j*. *x*_*ijt*_ is a vector of determinants of *y*_*ijt*_ while *β* is a vector of regression coefficients which show the effect of *x*_*ijt*_ on *y*_*ijt*_. The variable *ε*_*ijt*_ represents all other individual level determinants of *y*_*it*_ that we are not able to observe. It is normally distributed with mean zero and variance, $\sigma_{\varepsilon_{ijt}}^{2}$. Similarly, *μ*_*jt*_ represents all other community level unobservable determinants of child *i*’s outcome. It has mean zero and variance, $\sigma_{\mu_{jt}}^{2}$. If variation at the community level, $\sigma_{\mu_{jt}}^{2}$, is sufficiently small—approaching zero—then multilevel modelling is not necessary. We test the hypothesis that community level factors are not important determinants of childhood ill-health by assessing the size and significance of the intra-cluster correlation (ICC). The ICC is given as:

$$ICC=\frac{\sigma_{\mu_{jt}}^{2}}{\sigma_{\mu_{jt}}^{2}+\;\sigma_{\varepsilon_{ijt}}^{2}}$$ (3)

This paper does not aim to conduct a full multilevel analysis. Our only interest is to see whether or not, broadly viewed, the community in which a child lives matters for their health. As such, no covariates are included at the second level. We are only interested in the ICC and the coefficients in *β*.

The above regression model can be estimated using multilevel logistic regression since *y*_*ijt*_ is binary. Our interest is to also use the coefficients in *β* in the decomposition of the concentration index. However, since logistic regression is nonlinear while the decomposition of the concentration index requires linearity, we can either compute partial effects (probabilities) from the log odds, *β*, or use the log odds themselves in the decomposition.

Partial effects have the advantage of being easily understood. However, generating them from the vector *β* in multilevel logistic regression is complicated. Since we are interested in partial effects, and for ease of interpretation as well as computation simplicity, we used the multilevel linear regression which yields direct estimates of partial effects. Linear regression as a method of modelling binary variables, formally termed linear probability models (LPM), has seen widespread use in the literature lately and yields partial effects that are not different from probit or logistic regression partial effects [30–32]. It has been shown that if interest is not in prediction but simply the coefficients vector, *β*, then the LPM is very appropriate [33].

### Inequality in stunting and fever

We use the concentration index to quantify the extent of socio-economic inequality in the prevalence of stunting and incidence of fever in 2007 and 2014. The concentration index summarizes the extent to which good or bad health is dependent on income or wealth and it may be explained using the concentration curve concept. The concentration curve plots the cumulative share of health (on the y-axis) against the cumulative proportion of the population, ranked by wealth, from poor to richest (on the x-axis). For example, the concentration curve may show the cumulative percentage of stunting accruing to the poorest 25% of the population. To be complete, suppose that we want to look at inequality in ill-health. If the concentration curve lies on the 45-degree line, then the cumulative share of ill-health is equally shared between the rich and the poor and there is no socioeconomic inequality in health. However, if the concentration curve lies on the left of the 45-degree line, then the poor carry a disproportionately high share of ill-health.

The standard concentration index is twice the area between the concentration curve and the 45-degree line and in any given year, *t*, it can be written as:

$$CI_{y_{t}}=\frac{2}{N{\bar{y}}_{t}}\sum_{i=1}^{N}y_{ijt}R_{ijt}-1$$ (4)

where $\bar{y_{t}}$ is the average rate of fever or stunting in year *t*. *R*_*ijt*_ is the rank of child *i*’s household in the wealth distribution, in our case measured by the wealth index from principal component analysis. The concentration index ranges from -1 to 1. It is zero if there is no socioeconomic inequality in health, -1 if all the ill-health is borne by the poor, and +1 if the richest have all the ill-health. It has been shown however that the concentration index may not be bounded between -1 and +1 if the health variable is binary [34], as it is in our case. This may lead to misleading conclusions. In particular, the bounds of the concentration index for a binary variable depend on average health and this can cause problems if one is comparing inequalities for two different areas or time periods that have substantially different average levels. This is important in our case since we compare inequality between 2007 and 2014.

Two alternative normalizations of the standard CI have been proposed by Wagstaff [34] and Erreygers [35]. The standard CI is a measure of relative inequality, which is also the emphasis of the Wagstaff normalization. On the other hand, the Erreygers normalization is an absolute measure. It has been shown that neither of the two normalizations is superior to the other but each of them embodies different value judgements [36]. We used the Wagstaff normalization in this paper. The normalization involves dividing the standard concentration index in Eq 4 by $\left(1-\bar{y_{t}}\right)$ which give:

$$CI_{y_{t}}=\frac{\frac{2}{N{\bar{y}}_{t}}\sum_{i=1}^{N}y_{ijt}R_{ijt}-1}{\left(1-\bar{y_{t}}\right)}$$ (5)

For each outcome, we computed this index in 2007 and 2014 to assess the extent of inequality in each year.

### Changes in overall concentration index

For each outcome, *y*, we computed the change in the concentration index as follows;

$$\Delta CI_{y}=CI_{y_{2014}}-CI_{y_{2007}}$$ (6)

The computation of the normalized CI based on Eq 5 and the change in the index as specified in Eq 6 involves a four stage computation process, which raises the issue of how to appropriately compute confidence intervals. In estimating the normalized CI for each year, the first stage involves the computation of the mean of the outcome and weighted fractional wealth rank for each year. In the second stage, these estimates are combined to estimate the standard CI. The third stage involves dividing the standard CI by $\left(1-\bar{y_{t}}\right)$ to obtain the normalized CI. The change in the concentration index adds a fourth step to these computations; subtracting the 2007 normalized CI from that of 2014.

Our challenge is that since each estimate in these stages is computed from survey data, it has uncertainties which have to be taken into account when computing standard errors. Using analytical standard errors (from the last stage only) would make confidence intervals appear narrower than they actually are. To guard against this problem, we employ a bootstrap procedure with 1,000 replications. This involves repeating the above four step procedure 1,000 times, each time collecting the estimates, and then using these estimates to compute confidence intervals—which are then called bootstrap confidences intervals.

### Decomposing changes in the concentration index

To decompose the changes in the overall concentration index, we make use of the estimated partial effects of determinants of fever/stunting, *β*, from Eq 1. The concentration index for outcome *y* in year *t* can then be written as a sum of the weighted concentration indices for all the determinants of *y* plus the generalized concentration index for the error term:

$$CI_{y}{}_{{}_{t}}=\sum_{k}\left(\frac{{\hat{\beta }}_{kt}{\bar{x}}_{kt}}{{\bar{y}}_{t}}\right)CI_{kt}+\frac{GC_{\varepsilon t}}{{\bar{y}}_{t}}$$ (7)

where *CI*_*kt*_ is the concentration index for determinant *k* at time *t* computed as in Eq 5, that is, *y*_*ijt*_ in Eq 5 is replaced with *x*_*ijt*_ to get *CI*_*kt*_. The weight, $\left(\frac{{\hat{\beta }}_{kt}{\bar{x}}_{kt}}{{\bar{y}}_{t}}\right)$ is the elasticity of the *k*^th^ variable with respect to the health variable *y*_*ijt*_ at time *t* and *GC*_*εt*_ is the generalized concentration index for the error term. *GC*_*εt*_ is obtained by multiplying the concentration index for the error term by the mean of the outcome, ${\bar{y}}_{t}$. Thus, $\frac{GC_{\varepsilon t}}{{\bar{y}}_{t}}$, is the concentration index for the error term. At any given time, *t*, Eq 7 says that the concentration index of *y*_*t*_ can be written as a weighted sum of the concentration indices of the *K* determinants plus the concentration index of the unobserved determinants of *y*_*t*_. The weight for each concentration index of the determinant, *CI*_*kt*_, is the elasticity of *y*_*t*_ with respect to that determinant (note that the elasticity is a nonlinear combination of ${\hat{\beta }}_{kt}$, ${\bar{x}}_{kt}$ and ${\bar{y}}_{t}$).

Eq 7 is the most commonly used method of decomposing inequalities in child health. Clearly this decomposition only allows one to examine the relative contribution of various determinants in explaining inequality at any given time, but it does not allow one to see which determinants are driving changes in inequality at any two given periods. To examining the drivers of changes in the childhood ill-health inequality specified in Eq 6 we apply the Oaxaca decomposition to Eq 7 [26]. This leads to the following:

$$\Delta CI_{y}=\sum_{k}\eta_{k2014}\left(CI_{k2014}-CI_{k2007}\right)+\sum_{k}CI_{k2007}\left(\eta_{k2014}-\eta_{k2007}\right)+\Delta \left(\frac{GC_{\varepsilon t}}{{\bar{y}}_{t}}\right)$$ (8)

where *η*_*kt*_ is the elasticity of *y* with respect to determinant *k* in year *t*. Since $\;\eta_{kt}=\left(\frac{{\hat{\beta }}_{kt}{\bar{x}}_{kt}}{{\bar{y}}_{t}}\right)$, the elasticity of determinant k, *η*_*kt*_, can change due to changes in any of its component, namely, ${\bar{y}}_{t}$, ${\hat{\beta }}_{kt}$, and ${\bar{x}}_{kt}$.

Eq 8 says that changes in the concentration index of health outcome *y* can be written as a sum of three components, namely, the weighted sum of the changes in the inequality of the *K* determinants, the weighted sum of the changes in the elasticities of *y* with respect to the *K* determinants, and the change in inequality of unobservable determinants. The change in inequality of each determinant is weighted by the elasticity of *y* with respect to this determinant in 2014 while the change in elasticity is weighted by the inequality of the determinant in 2007.

In other words, apart from the contribution of unexplained factors, $\Delta \left(\frac{GC_{\varepsilon t}}{{\bar{y}}_{t}}\right)$, the contribution of the *k*^th^ determinant to the change in inequality in *y*, Δ*CI*_*y*_, can be brought about by the change in the concentration index of the k^th^ determinant, (*CI*_*k*2014_ − *CI*_*k*2007_), or the change in it’s the elasticity, (*η*_*k*2014_ − *η*_*k*2007_), or both. An increase in the concentration index of the *k*^th^ determinant in 2014 increases its contribution to inequality. On the other hand, the increase in its elasticity in 2014—resulting from a change in ${\bar{y}}_{t}$, ${\hat{\beta }}_{kt}$, ${\bar{x}}_{kt}$ or any other combination of these—can also contribute to the increase in inequality of childhood ill-health. For example, consider a case where the *k*^th^ determinant is concentrated on the well-off (*CI*_*kt*_ > 0) and it has a protective effect (${\hat{\beta }}_{kt}$ is negative). In this case, a reduction in the prevalence of *y*, the mean ${\bar{y}}_{t}$, will increase inequality in *y*. Similarly, an increase in the mean of the *k*^th^ determinant, ${\bar{x}}_{kt}$ will increase inequality. Holding ${\bar{y}}_{t}$ and ${\bar{x}}_{kt}$ constant, an increase in ${\hat{\beta }}_{kt}$ will also increase inequality.

## Results

### Descriptive statistics

The characteristics of children, mothers and households changed between the years 2007 and 2014. There was a substantial and significant increase in the proportion of children being delivered at a health facility in 2014 (Table 1). Birthweight was slightly lower in 2014 but the average duration of breastfeeding remained the same in both periods.

**Table 1:** Descriptive statistics.

Variable	Mean		P-Value (Differences)
	2007 (N = 5,61)	2014 (N = 12,714)	H_0_: Mean_2007_ = Mean_2014_
**Child’s Characteristics**			
Delivered at facility (%)	46.5	67.7	0.00
Birthweight (grams)	3238.1	3186.9	0.00
Childs age (months)	27.6	28.8	0.00
Duration of Breastfeeding (Months)	15.9	16.0	0.69
Birth Order	3.8	3.8	0.35
**Mothers’ Characteristics**			
No Education (%)	13.7	11.1	0.00
Primary Education (%)	63.7	56.3	0.00
Secondary Education (%)	20.4	29.0	0.00
Higher Education (%)	2.2	3.6	0.00
Height (cm)	157.3	157.6	0.020
Weight (kg)	55.5	56.5	0.00
Age (years)	28.6	28.9	0.00
Employed (%)	59.4	59.1	0.78
**Households’ Characteristics**			
Rural (%)	71.5	66.3	0.00
Improved Water Source (%)	35.7	59.5	0.00
Improved Toilet (%)	17.7	22.5	0.00
Household Size	6.2	6.5	0.00
Number of Children below 5 years	1.99	1.95	0.02
			

In 2014, mothers’ education levels generally improved with significantly more mothers having secondary or higher education. Mothers of children under the age of five were also slightly larger in size -in term of height and weight- in 2014 and were also slightly older.

Living conditions also changed. The proportion of children coming from rural households was significantly lower in 2014. Access to improved sources of water increased substantially as did the proportion with improved toilets, although this increase was not as substantial.

There was no practically significant difference in household size and the number of under-5 children in the two periods.

### Regression results

#### Clustering within communities

Table 2 shows two level random intercept models for stunting and fever by survey year. The intra cluster correlation for both stunting and fever are significantly different from zero implying that there is significant clustering of both stunting and fever. However, this clustering is higher for fever than it is for stunting.

**Table 2:** Effect of different factors on the probability of stunting and fever by year.

	Stunting		Fever	
**Variable**	2007	2014	2007	2014
**Wealth Quartile**				
Quartile 1 (Poorest)	Base	Base		
Quartile 2	0.042 ((-0.023)—(0.106))	-0.032 ((-0.066)—(0.002))*	0.009 ((-0.020)—(0.039))	0.003 ((-0.019)—(0.024))
Quartile 3	0.031((-0.035)—(0.098))	-0.016 ((-0.053)—(0.021))	0.006((-0.028)—(0.040))	-0.018 ((-0.043)—(0.006))
Quartile 4 (Least poor)	-0.030((-0.114)—(0.053))	-0.042 ((-0.087)—(0.004))*	0.012((-0.038)—(0.063))	-0.034 ((-0.067)—(-0.002))**
**Maternal Education**				
No Education	Base	Base	Base	Base
Primary	0.008((-0.064)—(0.081))	-0.000 ((-0.040)—(0.040))	0.014((-0.018)—(0.045))	-0.028 ((-0.052)—(-0.003))**
Secondary	-0.016((-0.096)—(0.064))	-0.014 ((-0.058)—(0.031))	-0.002((-0.042)—(0.037))	-0.027 ((-0.057)—(0.002)) *
Higher	-0.038((-0.162)—(0.085))	-0.069 ((-0.138)—(-0.001))**	-0.042((-0.125)—(0.041))	-0.057 ((-0.109)—(-0.005))***
**Other Maternal characteristics**				
Age	-0.001((-0.007)—(0.004))	-0.004((-0.007)—(-0.001))***	-0.001((-0.004)—(-0.002))	-0.004((-0.006)—(-0.002))***
Height	-0.009((-0.012)—(-0.006))***	-0.010 ((-0.012)—(-0.008))***		
Weight	-0.002((-0.004)—(-0.000))**	-0.002((-0.003)—(-0.001))***		
Employed	-0.020((-0.058)—(0.019))	0.017((-0.006)—(0.039))	0.045((-0.023)—(0.067))***	0.036((0.020)—(0.053))***
**Household Characteristics**				
Rural	-0.001((-0.062)—(0.061))	-0.026((-0.057)— (0.005))	-0.006((-0.049)— (0.037))	-0.007((-0.033)— (0.020))
Improved water source	-0.000((-0.047)—(0.046))	-0.007((-0.033)— (0.018))	-0.009((-0.037)— (0.019))	-0.015((-0.033)— (0.003))
Improved toilet	-0.041((-0.092)—(0.009))	0.002((-0.025)— (0.028))	-0.009((-0.041)— (0.023))	-0.000((-0.020)— (0.020))
Household Size	-0.008((-0.017)—(0.002))	-0.006((-0.011)—(-0.001))**	0.006((0.001)—(0.012))**	0.007((0.003)—(0.011))***
Number of Children below 5 years	0.035 ((0.009)— (0.061))***	0.012 ((-0.003)— (0.026))	-0.020 ((-0.035)—(-0.006))***	-0.032 ((-0.042)—(-0.022))***
**Child Characteristics**				
Born at Facility	0.022((-0.041)—(0.085))	-0.016((-0.060)—(0.029))	-0.014((-0.037)—(0.010))	0.026((0.008)—(0.044))**
Birth Weight (kg)	-0.088((-0.117)—(-0.058))***	-0.095((-0.113)—(-0.077))***		
Male	0.070 ((0.033)— (0.106))***	0.058 ((0.037)— (0.079))***	0.022 ((0.002)— (0.042))**	-0.004((-0.018)— (0.012))
Age	-0.000((-0.002)—(0.001))	-0.001((-0.002)—(-0.000))**	-0.003((-0.004)—(-0.002))***	-0.002((-0.003)—(-0.001))***
Duration of breastfeeding	0.012 ((0.009)— (0.015))***	0.012 ((0.010)— (0.013))***	0.005 ((0.003)— (0.007))***	0.006 ((0.005)— (0.007))***
Birth order	-0.002((-0.018)— (0.014))	0.016((-0.006)— (0.025))***	-0.005((-0.013)— (0.004))	0.006((-0.001)— (0.012))
	Statistics			
**Intra Cluster Correlation**	0.025 ((0.010)—(0.056))***	0.027((0.017)—(0.042))***	0.047((0.034)—(0.066))***	0.060((0.049)—(0.073))***

Table shows estimates and 95% confidence intervals (in parenthesis) from a multilevel linear probability model for stunting and fever for each year.

***Significant at 1%.

**Significant at 5%.

*Significant at 10%.

#### Factors associated with stunting

In both years, lower height and lower weight of the mother was associated with a higher likelihood of stunting, with height exhibiting a particularly strong relationship. High birthweight was also associated with lower likelihood of stunting in both years, while longer duration of breastfeeding and child being male were associated with higher likelihood of stunting.

Wealth and higher education level of the mother were associated with lower likelihood of stunting in 2014 but not in 2007. Similarly, in 2014, children of older mothers were less likely to be stunted than those with young mothers while children in higher birth order were more likely to be stunted.

#### Factors associated with fever

In both years, longer duration of breastfeeding was associated with higher likelihood of fever. A child whose mother was employed either in the formal or agricultural sector was more likely to experience fever compared to one whose mother was unemployed. Children from households that were larger were also more likely to have fever. Having a large number of children under 5 years in the household was associated with a lower likelihood of having fever. The likelihood of having fever is also lower the older the child.

As is the case with stunting, wealth and education were significantly associated with fever in 2014, but did not appear as important in 2007. In particular; any form of mothers’ education was associated with lower likelihood of fever.

### Socioeconomic inequality in childhood ill-health

Zambia had significant socioeconomic inequalities in stunting in both 2007 and 2014 (Table 3). The negative sign of the concentration indices indicates that children from poorer households carried a disproportionally higher burden of stunting than their relatively better-off counterparts. In spite of the reduction in stunting prevalence from 45.6% in 2007 to 40% in 2014, the levels of inequality as measured by the Wagstaff CI significantly increased by about 45%, from -0.093 in 2007 to -0.135 in 2014. The increase in the concentration index implies that stunting was reduced less among the poor. In fact, a tabulation of stunting levels by wealth quartiles (not reported) shows that the poorest quartile did not register any change in stunting.

**Table 3:** Mean levels and socioeconomic inequality in stunting and fever in 2007 and 2014.

	2007	2014	H_0_: Y_2007_ = Y_2014_
	Estimate (95% Bootstrap CI)	Estimate (95% Bootstrap CI)	Bootstrap P-Value**
**Prevalence of Stunting**			
Mean	0.456(0.442–0.471)	0.400(0.389–0.410)	0.000
Concentration Index	-0.093((-0.128)—(-0.058))	-0.135((-0.160)—(-0.109))	-0.041(0.051)
			
**Incidence of Fever**			
Mean	0.184(0.173–0.195)	0.216(0.208–0.225)	0.000
Concentration Index	-0.015((-0.057)—(0.027))	-0.064((-0.092)—(-0.036))	-0.049(0.055)
			

**Note that for the concentration indices, the p-values are in parenthesis

In 2007, there is no evidence of inequality in fever incidence (Table 3). Inequalities however, emerged in 2014, with a concentration index of -0.064. The incidence of fever also increased slightly from 18.4% to 21.6%.

### Decomposition of changes in concentration index

#### Explaining changes in the inequality of stunting

The concentration index of stunting increased by -0.041 (became more pro-poor—i.e., stunting was reduced less among the poor so that inequalities increased) between 2007 and 2014 (Table 3). This increase in inequality of stunting was accounted for by both the increase in the CI of determinants (42.5%) and the increase in the effect of determinants (35%), measured as elasticities (Table 4). The rest of the increase (22.5%) was due to unexplained factors.

**Table 4:** Decomposition of the change in inequality in stunting and fever.

	Stunting				Fever			
	Weighted Δ in CI	Weighted Δ in elasticity	Total Δ	Total % Δ	Weighted Δ in CI	Weighted Δ in elasticity	Total Δ	Total % Δ
**Wealth Quartile**								
Quartile 1 (Poorest)								
Quartile 2	0.001	0.009	0.010	-25	-0.000	0.002	0.001	-3
Quartile 3	0.001	-0.007	-0.006	16	0.001	-0.007	-0.006	11
Quartile 4 (Least poor)	0.001	-0.007	-0.007	16	0.001	-0.044	-0.043	88
**Maternal Education**								
No Education								
Primary	0.000	0.001	0.001	-3	0.006	0.009	0.015	-30
Secondary	0.000	-0.001	-0.000	0	0.001	-0.013	-0.011	23
Higher	0.000	-0.004	-0.003	9	0.000	-0.005	-0.004	9
**Other Maternal characteristics**								
Age	-0.003	0.002	-0.000	1	-0.005	0.004	-0.001	2
Height	-0.009	-0.001	-0.010	25				
Weight	-0.004	-0.000	-0.005	12				
Employed	0.000	-0.004	-0.004	9	0.001	0.002	0.002	-5
**Household Characteristics**								
Rural	0.001	0.011	0.012	-30	0.000	0.000	0.001	-2
Improved water source	0.003	-0.005	-0.002	6	0.009	-0.012	-0.003	5
Improved toilet	-0.000	0.010	0.009	-23	0.000	0.003	0.003	-7
Household Size	-0.001	0.000	-0.001	2	0.002	0.000	0.002	5
Number of Children below 5 years	0.000	0.004	0.004	-10	-0.001	0.003	0.002	-5
**Child Characteristics**								
Born at Facility	0.003	-0.014	-0.010	26	-0.010	0.030	0.019	-39
Birth Weight (kg)	-0.001	0.000	-0.001	3				
Male	-0.001	-0.000	-0.001	2	0.000	-0.001	-0.001	1
Age	-0.000	-0.000	-0.000	1	-0.001	0.000	-0.001	1
Duration of breastfeeding	-0.006	0.000	-0.005	13	-0.006	-0.002	-0.008	16
Birth order	-0.001	-0.010	-0.011	27	-0.000	-0.011	-0.012	24
Residuals			-0.009	24			-0.007	14
Total	-0.017	-0.014	-0.041	100	-0.000	-0.042	-0.049	100
Percent of total Δ*	42.5	35		100*	0	86		100*

Table 4 shows the decomposition of the change in CI according to Eq 8 for stunting (first 4 columns) and fever (next 4 columns). The total change is given in column 3 and 7 of the last but one row. The variables that contributed positively to this increase have a negative quantity in column 3 and 7 (negative because they made the CI more negative—increased concentration of ill-health on the poor). This translates to a positive percentage change in contribution to inequality (Column 4 and 8).

*This adds column 1 and 2, the difference is due to residuals.

The determinants that contributed most to the increase in inequality of stunting were mother’s height and weight (37%), being in the two wealthiest quartiles (32%), birth order (27%), facility delivery (26%), duration of breastfeeding (13%), and higher level of maternal education (9%). Other factors worked to reduce inequality and hence have negative percentage contribution to the increase in inequality. But how did the change in CI and effect of each of these determinants contribute to the increase in inequality of stunting? Table 4 shows that the CI for height and weight increased (became more pro-rich—heights and weights increased more for the rich) while at the same time the effect of these determinants on stunting increased. These two mechanisms reinforced each other to drive inequality in stunting up, with the increase in the CI having a particularly larger contribution. On the other hand, since the CI of wealth itself reduced, the contribution of wealth to the increase in inequality (in the top 2 quartiles) was solely due to the increase in the effect of wealth. The change in the CI of birth order and the change in the effect of birth order on stunting reinforced each other to drive inequality up. In particular, the increased effect of birth order on stunting was both a result of higher birth order becoming more significantly associated with increased likelihood of stunting in 2014 (Table 2) and a reduction in the prevalence of stunting (Table 3). At the same time, birth order became more concentrated among the poor implying that the poor bore a disproportionately larger share of the risk arising from higher birth order.

Since the CI of facility deliveries itself reduced (became less pro-rich—facility deliveries increased more among the poor), the contribution of facility deliveries to the increase in inequality of stunting was almost entirely driven by the increase in its effect on stunting. Despite the reduction in the CI, however, inequality in facility deliveries remained pro-rich (the rich still had higher access to facility deliveries). Hence the increase in the protective effect of facility deliveries disproportionately benefited the better off. The same can be said about maternal education; the effect strengthened but this benefit accrued more to the better off since they had a disproportionately larger share of higher education. The contribution of duration of breastfeeding to the increase in inequality was entirely due to the inequality effect; longer periods of breastfeeding becoming more concentrated on the poor.

#### Explaining changes in the inequality of fever

While both changes in the CI (inequality) of determinants as well as the effects (elasticity) of determinants were important in explaining increasing inequality of stunting, almost all (86%) the increase in the inequality of fever incidence was accounted for the change in the effects of determinants (Table 4). The changes in the CI of determinants, overall, did not explain any increase in the inequality of fever, implying that the rest of the increase—14%—was accounted for by unexplained/unobserved determinants. The key contributors to the increase in inequality in fever incidence were wealth (99%), mother’s education (32%), birth order (24%), and duration of breastfeeding (16%). Note that the overall contribution of all determinants add up to 100% because other determinants worked to reduce inequality (have negative percentage contribution)

As indicated earlier, the CI for wealth reduced slightly although it remained pro-rich. However, there was a substantial strengthening of the effect of wealth on fever in 2014, and due to the highly pro-rich distribution of wealth, most of this benefit accrued to the better off. This drove inequality in fever up, as did maternal secondary or higher education.

Even if almost all the increase in inequality of fever was accounted for by the increase in the effect of determinants, some determinants’ contribution were both due to the change in their effect and their concentration indices. This can be said of birth order whose contribution to the increase in inequality of fever was due to the two mechanisms reinforcing each other—higher birth order becoming more concentrated on the poor and a strengthening of effect of birth order on fever.

## Discussion

We investigated determinants of, and socioeconomic inequality in, stunting and fever in Zambia between 2007 and 2014, a period when child health interventions were rapidly scaled up to meet the 2015 MDG target on child health. We find that although stunting prevalence reduced, inequality increased. On the other hand, fever incidence did not fall but inequality still increased. The increase in inequality of stunting and fever implies that the rapid scale up of child health interventions may not have been successful in reducing childhood disease burden among the most vulnerable, suggesting the need for policy reform if the goal of reducing inequality, as captured by the Sustainable Development Goals (SDGs), is to be achieved.

We also find evidence of clustering for both stunting and fever implying that the likelihood of being stunted or having fever partly depends on the area in which the child lives, which is particularly apparent for fever. Elsewhere, fever has also been shown to exhibit substantial clustering [37–39], and in many African countries, it is highly associated with malaria and pneumonia [40]. Clustering is a form of inequality and implies that some areas suffer higher burden of childhood ill-health than others.

Our study included a rich set of determinants that potentially explain the likelihood of a child getting fever or being stunted. We document a very strong association between maternal size (height and weight) and stunting. The association between maternal height and weight on one hand and stunting on the other hand may be due to genetic factors or maternal nutritional deficiencies—showing up as low maternal weight and short maternal height. Maternal nutritional deficiencies may lead to in-utero growth restriction, [41], so that children whose growth was restricted end up being stunted. However, while low maternal weight may be directly related to nutrition during pregnancy which directly impacts on fetal growth, maternal height is related to nutrition during the mother’s own childhood and only affects fetal growth indirectly through, for example, smaller sizes of reproductive organ, reduced protein and energy stores, and limited room for child development in utero [42–44]. Our findings are consistent with a multi-county randomized trial that included Zambia [45]. Since there is a strong correlation between maternal height, weight and social economic status, our results imply that previous studies investigating inequality in stunting that did not control for maternal height and weight may have overestimated the effect of socioeconomic variables such as wealth and education.

In line with the strong and consistent correlation, the decomposition analysis showed that inequality of maternal height and weight was the biggest driver of the increase in inequality in stunting over the period 2007–2014. The increase in inequality was mainly due to the fact that more advantageous heights and weights became more concentrated on wealthier mothers. It is therefore important to reduce inequality in maternal nutrition, both early in life and during pregnancy, to halt increases in inequality in stunting.

Another interesting finding relates to birth order. We found that higher birth order was a risk factor for stunting and fever. These findings are consistent with a number of studies that have documented a negative association between higher birth order and child health [46–49], education attainment [50–54] as well as cognitive abilities [55]. Debate on the exact mechanism through which higher birth order is a risk factor for most outcomes seems to be polarized with others indicating that the cause is biological and others indicating that it is confounded by family size, a variable we control for in our set-up. Yet others have pointed to the social interaction mechanism where children born later receive less favorable social interactions. Consensus seems to have emerged that the social interaction mechanism is the cause of the observed association [55, 56]. We have documented that birth order contributed to the increase in inequality of both stunting and fever as higher birth orders became even more concentrated among the poor. If the social interaction hypothesis is true, it may be beneficial to use routine health programs to emphasize the importance of child care for children of higher birth orders.

We also document a consistent correlation between duration of breastfeeding and childhood ill-health where possible confounders, including wealth, are adjusted for. In results not reported, this correlation was generally maintained in all households after stratifying by wealth quartile. There is mixed evidence on the effect of breastfeeding duration on child health. A number of studies find a positive correlation between duration of breastfeeding and poor growth [57–62] while others do not. It is generally held that this positive correlation is due to two possible mechanisms. First is the case of reverse causality were children who are in poor health to begin with are breastfed for longer. We cannot rule out this possibility since we are using cross sectional data. Note however that the relationship between breastfeeding duration and childhood ill-health in our set up is not likely to be driven by differences in wealth, maternal education, maternal nutrition, maternal age, child’s birthweight, etcetera, because we control for these possible confounders in the regression analysis. Second, there is possibility that sufficient complementary food is not provided to meet energy and nutritional demands of the child [63]. The insufficient feeding argument seems compelling given that longer breastfeeding duration, while possibly having other benefits, may not be helpful for child growth without sufficient complementary feeding [64–66]. Proteins, necessary for growth, may become deficient if there is laxity in providing adequate complementary food and more emphasis is placed on breastfeeding [65]. If we are comparing children of the same birthweight, age, sex and coming from equally wealthy households, then laxity in providing complementary feeding may explain the observed relationship.

Although longer breastfeeding duration was a risk in all wealth quartiles, poorer households breastfed longer than their well off counterparts. The concentration of longer breastfeeding duration on poorer households contributed to the increase in inequality of childhood illness. One should however, interpret these results with caution because breastfeeding in itself has been shown to have other beneficial effects [67]. Perhaps what comes out from this finding is that programs should emphasize sufficient complementary finding even with longer breastfeeding to ensure sufficient protein and energy intake. It has been shown that even if children from low income countries, in general, start at the same average height for age as the reference population, there is rapid faltering of growth in the first 2 years of life [68, 69]. This faltering may suggest inadequate quality and quantity of complementary foods. However, disease may also explain this poor growth given a complex interaction between malnutrition and disease.

Facility deliveries may be an important entry point for feeding counselling and support interventions. This assertion is supported by evidence from Uganda were being delivered at a facility was associated with better child feeding practices and nutritional status of children [70]. We find that the increase in the effect of facility deliveries (in terms of elasticity) contributed to the increase in inequality of stunting (driven by the increase in mean level of facility deliveries, reduction in mean stunting and improvement in the association between facility delivery and stunting). However, increases in facility deliveries mainly benefited the well off more because they were unequally concentrated on the well off to begin with. Thus, the increased effect also contributed to the increase in the inequality of stunting.

The question emerges, how can policy halt the increasing socioeconomic inequalities in stunting prevalence and fever incidence?

One possible option would be to reduce, and possibly eliminate, the effect of determinants that increase the risk of stunting and fever. This can be done in three ways, as can be seen when the elasticity formula is unpacked (we discussed this mechanism in the last part of the methods section). First, the average level of determinants that are risk factors for child health can be reduced. Second, the association (marginal effect) between (of) the determinant and (on) child health can be eliminated. For example, in the case of birth order, routine health education programs may emphasize the importance of giving as much attention to children of higher birth order as those of lower birth order. This may diminish the association and thus reduce inequality. Third, and lastly, the incidence or prevalence of the relevant childhood ill-health can be reduced.

The other option would be to reduce inequality (concentration indices) in (of) determinants such as wealth, education, maternal nutrition, etcetera, which are protective for child health. These determinants are unequally concentrated on the well off and reducing their inequality may be beyond the scope of health interventions. It is important to realize that health interventions can mainly affect the effect, in terms of elasticity, of these determinants on child health but not their distribution. For example, since more educated mothers are likely to understand and follow health instructions better, health interventions such as infant and child feeding, breastfeeding counselling, etc., may increase the association between maternal education and stunting but are not able to reduce inequality in education. The same can be said about wealth. Therefore, it is easy to see why, despite the rapid increase in child health intervention coverage (such as infant and child feeding counselling and support) and the accompanying increase in the effects of determinants, inequality in childhood-ill-health still increased. The dichotomy is that increasing the effect of determinants with protective effects, which is a good thing in general, worsens inequality if these determinants are disproportionately concentrated on the rich in the first place. This is an example of the classical equity-efficiency trade off. Despite the persistence of inequality, other studies have documented substantial improvements in other measures of child health, such as under-five mortality [24, 71]. Our findings suggest that one of the reasons for the persistent inequalities is that health improvements disproportionately benefit the well off because the determinants of childhood good health such as access to health facilities, which health and other interventions seek to improve, are unequally concentrated on the well-off, to begin with.

Our study has limitations and due caution must be excised when interpreting the findings. It must be noted that no community level covariates were included at the second stage of the multilevel model. This raises a possibility of confounding if the omitted community level covariates are correlated with both childhood ill health (stunting or fever) and any included individual or household level variable. Moreover, being based on cross-sectional data, our results cannot be viewed as causal.

It is worth mentioning, however, that the data we have used, the Demographic and Health Survey (DHS) presents both limitations and strengths for inequality analyses. It is a limitation because DHS relies on wealth indices and does not contain finer measures of household living standards, such as consumption or income. Income or consumption has the advantage of being able to be objectively measured and compared across different places or surveys with less difficulty. Wealth indices may be problematic if one is comparing two populations (e.g rural with urban or population in 1970 with population in 2014) as the type of assets and their valuation may differ across populations and time. Although some methods on how to make wealth indices collected in two different populations or two different points of time have been proposed [72], they are, at best, imperfect. To reduce this comparability problem, analyses, and concentration indices were calculated separately for each year.

Using the DHS is also a strength of our study. This is because it contains a rich set of health variables. As an alternative, we could have followed studies from Vietnam and used data from the Living Conditions Measurement Surveys(LCMS)which have information on income, consumption and anthropometric measurements. However, the LCMS does not contain a rich set of health variables, as does the DHS, and this can potentially confound the relationship between key socioeconomic variables and child health. It also does not contain other child health outcomes such as fever.

## Conclusion

Childhood ill-health has serious consequences. Apart from increasing under-5 mortality rates, it negatively affects cognitive abilities, education attainment, later life income, and adult health. However, children in low socioeconomic background bear a significantly larger share of childhood ill-health implying that they will continue to shoulder a larger share of these adverse consequences. This raises ethical issues. Why should children from poor backgrounds experience more ill-health when the determinants of ill-health are beyond their control, and to a large extent beyond the control of their parents. How can such inequalities be justified when they are hugely generated by inequality of opportunities to determinants of good health, such as education and health care? Against this backdrop, reducing inequality constitutes one of the most important development goals and is now part of the post 2015 development agenda, the Sustainable Development Goals (SDG), the successor to the Millennium Development Goals (MDG). To derive lessons for the post 2015 agenda of designing interventions that are effective in improving overall child health and reducing inequality, it is important go beyond asking whether or not inequalities increased by undertaking an in-depth analysis of the forces that drive inequality.

We examined the determinants of stunting and fever using the 2007 and 2014 Zambia DHS data to explore the existence of socioeconomic inequalities in childhood ill-health indicators, and whether this inequality changed over the period. Most importantly, we quantified how changes in inequality of determinants and the changes in elasticity of stunting and fever with respect to their determinants could have contributed to the change in inequality between 2007 and 2014.

While the prevalence of stunting reduced substantially, inequality increased between 2007 and 2014. In fact, inequalities were worsened by the fact that the prevalence of stunting was reduced in all quartiles, except the poorest. This increase in inequality was largely a result of the increase in the inequality of factors that are associated with stunting. These factors include maternal height and weight, wealth, birth order, breastfeeding duration, facility deliveries and maternal education. Although the responsive of stunting to most of these factors, e.g. facility deliveries, increased in 2014, this benefit mostly accrued to the better off because the factors remained concentrated on the better off. As a consequence, the improved responsiveness of stunting to its determinants also contributed to the increase in inequality.

Regarding fever, almost all the increase in the inequality was account for by the increase in the responsiveness of the disease to the factors that determine it. By far the biggest driver of this change was wealth, then maternal education, birth order and breastfeeding duration.

The key message in this study is that halting the increase in the inequality in childhood ill-health depends heavily on reducing inequality in the factors that affect childhood ill-health while at the same time improving the impact (elasticity) of these factors using both health and non-health interventions. It is important to note that although improving impact of factors that affect child health is desirable, this in itself can be a source of increase in inequality if the factors whose impact are being improved are unequally concentrated on the better off to begin with.

Halting the increase in child health inequality is dependent on reducing inequality in the factors associated with child health. These include wealth, maternal education, appropriate feeding and weaning (related to breastfeeding duration effects), adequate care giving (related to birth order effects) and maternal nutrition (related to maternal height and weight). We believe that a more sustainable way of doing this is to ensure equality of opportunities in access to these factors among children from different socioeconomic backgrounds—who are future parents. This may call for policies that delink the dependence of child health on parental circumstances and the community they live. Specifically, group specific interventions aimed at the most vulnerable may need to be implemented along with population level interventions. For example, under the current social cash transfer scheme in Zambia, policy may aim at providing more cash benefits to the poorest households who have children under the age of five years. Poor households may also have special educational needs. For example, children from poor backgrounds may have challenges learning and concentrating even when given access to school due to persistent hunger. Child school feeding programs may help in improving attendance among poor children and also reducing inequality in learning and concentration.

Moreover, despite the fact that all households require appropriate breastfeeding and weaning educational interventions—due to the observed association between duration of breastfeeding and child health—poorer households may require special interventions since they have disproportionately longer breastfeeding durations. In general, the propensity to breastfeed longer may be due to lack of appropriate food or knowledge on how to use existing traditional food stuffs. Thus, policy may focus on introducing and scaling up complementary feeding and nutritional programs among poor households. Additionally, since the community in which a child lives matters for child health, policy may also focus on improving living conditions in disadvantaged communities, e.g. sanitation facilities, water, child care centers, etc. The implementation of such group specific intervention may enhance equality of opportunity and halt the increase in child health inequality.
